# Translation of professionalism in physical therapy questionnaire for Portuguese of Portugal: Core values

**DOI:** 10.1080/07853890.2021.1896840

**Published:** 2021-09-28

**Authors:** Mariana Carvalho, Sofia Mateus, Fábio Pinto, Inês Ramos, Sónia Vicente, Ângela Maria Pereira

**Affiliations:** a Department of Physiotherapy, Escola Superior de Saúde Egas Moniz (ESSEM) , Egas Moniz Cooperativa de Ensino Superior, Caparica, Portugal; b Centro de Investigação Interdisciplinar Egas Moniz (CiiEM), Egas Moniz Cooperativa de Ensino Superior, Caparica, Portugal; c Hospital Garcia de Orta, Almada, Portugal

## Abstract

**Introduction:**

Physiotherapists play a key role in today’s healthcare and are recognised as the professionals in charge of rehabilitation, prevention and risk reduction [1]. Professionalism is interconnected and inherent with the profession; physiotherapists have the duty to act in an ethically correct way and for this must be governed by certain ethical principles [2]. The evaluation of professionalism is difficult to achieve because of various qualities that should be present, and also because it is needed to use several measurement methods, particularly in complex real world contexts [3]. The linguistic adaptation of an instrument is carried out using the Delphi Method, which brings a group of experts where a series of rounds is conducted until consensus of the translation is reached [4]. The aim of the study is the translation of the instrument Professionalism in Physical Therapy: Core Values, of the American Physical Therapy Association (APTA), with the intention to be used by physiotherapists in Portugal.

**Materials and methods:**

The translation/back-translation was made by the Delphi Method with three rounds which were performed until de adaptation of the instrument to the Portuguese culture was achieved. A group of five experts, all with master and PhD academic degree, all with more than 25 years as Physiotherapist and Portuguese as their first language were selected.

**Results:**

In order to reach consensus on the translation of the instrument, 3 rounds were carried out using the Delphi method, thus allowing linguistic equivalence to be obtained. In the 1st round of the process regarding the physical therapist's part, a 92 questions questionnaire was sent to the experts. In the 2nd round 42 questions were held and in the 3rd and final round, 5 questions were translated. Regarding the patient's part of the questionnaire, in the 1st round 18 questions were sent and in the 2nd round the number decreased to 9 questions. Consensus was achieved at 2nd round. The level of minimum agreement in each item was set to 80%, ensuring good translation and adaptation for Portuguese culture.

**Discussion and conclusions:**

The aim of the study was achieved, and the linguistic translation was completed. As next step, it is needed to conduct validation of the instrument for Portuguese culture by performing a pre-test. The results of this content validation by experts will be useful to improve the standard of care, teaching and research in physiotherapy, based on a standardised instrument which allow to measure Portuguese Physiotherapists professionalism, and perform international benchmark as well.
